# COVID-19: A novel burden on the fragile health system of Angola

**DOI:** 10.7189/jogh.11.03059

**Published:** 2021-04-03

**Authors:** Dawa Gyeltshen, Shuaibu Saidu Musa, Josephine Ndapewoshali Amesho, Samuel Chukwuebuka Ewelike, Alex Vandy Saffa Bayoh, Christophe Al-Sammour, Angel Anthony Camua, Xu Lin, Mat Lowe, Attaullah Ahmadi, Blaise Ntacyabukura, Yusuff Adebayo Adebisi, Don Eliseo Lucero-Prisno

**Affiliations:** 1 Eusa Hospital, Ministry of Health, Wangdue Phodrang, Bhutan; 2 Department of Nursing Science, Ahmadu Bello University, Zaria, Nigeria; 3 Ministry of Health and Social Services of Namibia, Windhoek, Namibia; 4 Faculty of Pharmaceutical Science, Enugu State University of Science and Technology, Enugu, Nigeria; 5 HIV Program, ICAP Columbia University, Sierra Leone, Freetown, Sierra Leone; 6 Faculté de Médecine, Université Saint-Joseph de Beyrouth, Beirut, Lebanon; 7 College of Education, University of the Philippines Diliman, Quezon City, Philippines; 8 Department of Thoracic Surgery, School of Medicine, Zhejiang University, Hangzhou, China; 9 Society for the Study of Women’s Health (SSWH), Kombo North District, The Gambia; 10 Medical Research Center, Kateb University, Kabul, Afghanistan; 11 Global Public Health Department, Karolinska Institutet, Stockholm, Sweden; 12 Faculty of Pharmacy, University of Ibadan, Ibadan, Nigeria; 13 Department of Global Health and Development, London School of Hygiene and Tropical Medicine, London, UK; 14 Faculty of Management and Development Studies, University of the Philippines (Open University), Los Baños, Laguna, Philippines

Angola is a Sub-Saharan African country sharing borders with Namibia, Zambia and the Democratic Republic of Congo. COVID-19 has hit Angola at a time when it is grappling to heal long years of economic hardships fueled by dropping oil prices. Angola documented the first case of COVID-19 on March 21, 2020 [1]. As of January 18, 2021, 18875 COVID-19 cases have been confirmed with a recovery rate of 86.6% (16347) and a case fatality rate of 2.31% (436) [2]. The incidence rate of COVID-19 is 57.43 (per 100 000 people) which is lower than its neighboring countries such as Namibia, Botswana and Zambia [2]. Angola started to prepare its national contingency plan in February 2020 for the management of COVID-19, even before it reached the country [1]. Angola’s approach to dealing with COVID-19 is multisectoral - forming partnerships and cooperation with both local and international partners [3]. This paper aims to describe Angola’s responses and its preparedness against the COVID-19 pandemic.

## EARLY EFFORTS AND RESPONSES

A high level multidisciplinary taskforce for COVID-19 response was established that instituted a multisectoral response plan involving 23 key institutions co-chaired by the state, health and interior ministers [1]. Even before the first cases were detected, the Angolan government began quarantining returnees from COVID-19 affected countries [3]. A swift response to protect its health system was included but not limited to: quarantine, social distancing, closure of borders with limited exceptions, suspension of schools, restaurants, and public events, and limited transportation [4]. Since the first cases were detected, Angola ramped up education, testing and contact-tracing under its “National Contingency Plan to Manage the Pandemic” [3]. To ramp up its testing capacity, the country established Viana Laboratory Diagnostic Center with a testing capacity of 6000 samples per day in addition to other existing testing facilities [5].

A state-of-emergency was announced on 27 March 2020 imposing a two-week lockdown which was reviewed and extended on multiple occasions [6]. With this came stricter restrictions on public movements, closures of borders and public events and places among many others [7]. Although these restrictions helped keep COVID-19 cases low, it also proved to be a problem for families living on daily small earnings because their ability to earn such profits were affected [8] and on top of that Non-Governmental Organizations (NGOs) were unable to help the affected families due to restrictions imposed under the state-of-emergency [3]. On May 26, 2020, the state-of-emergency was scaled down to the state-of-calamity easing restrictions and allowing activities related to the functioning of the local economy whilst imposing some COVID-19 safety protocols [7]. Some of the non-health strategies to combat the pandemic included fiscal measures such as value-added-tax exemptions and regulation of drug prices; 30% freeze of government spending on non-essential goods and services; and monetary measures such as temporary suspension of debt payment among others [4].

Creation of multilateral partnerships in Angola’s fight against COVID-19 has been critical because its health system is under-resourced and its economy is in a desperate state. The Ministry of Health of Angola with the support of the World Health Organization (WHO) has deployed public health experts from Luanda to other provinces to train health professionals on monitoring and preventing COVID-19 [3]. The United Nations Children’s Fund (UNICEF), the United Nations Development Program (UNDP), the world bank, the United States Agency for International Development (USAID), and the International Monetary Fund (IMF) are some of the international partners that have assisted Angola in terms of both financial and technical support. The United States government and the European Union have both provided financial assistance to Angola to improve its testing capacity, provide training programs, enhance contact tracing and provide assistance to vulnerable populations [3]. Cuba sent doctors and medical supplies while Qatar and Portugal provided personal protective gears. Moreover, private partner companies such as ExxonMobil, Chevron and Jack Ma Foundation have also reached out to Angola donating personal protective equipment, and funding training programs among others [3].

**Figure:**
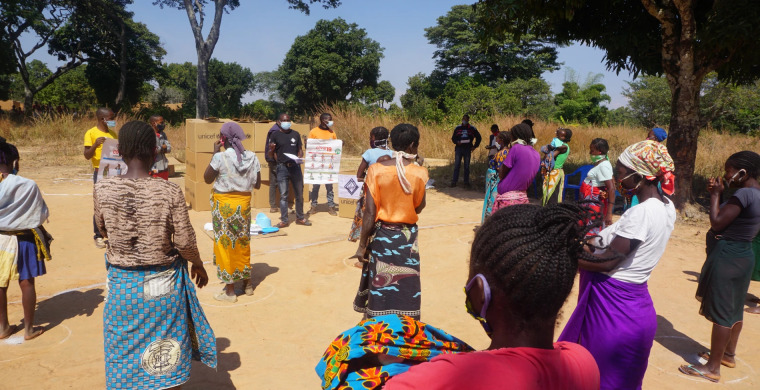
Photo: Vulnerable families gaining support in Angola amidst the COVID-19 pandemic. From: https://www.clovekvtisni.cz/en/supporting-vulnerable-families-in-angola-during-the-covid-19-pandemic-6779gp (Copyright free).

## CHALLENGES AND OBSTACLES

The under-resourced health system, high levels of poverty, limited social security, limited access to health, high unemployment rates among youth and women, non-compliance to the restriction measures among others are the obstacles that the Angolan government face in the fight against COVID-19 [9]. Misinformation and rumors about COVID-19 relating mainly to “mentions of disease, testing, treatment, vaccine and response by local authorities and partners” also pose a challenge. In July, 2020, Ministry of Health and the WHO in Angola set up a COVID-19 alliance-a system aimed at managing hoaxes and misinformation about COVID-19 [10]. Insufficient personal protective equipment (PPE) has led to health workers being infected with COVID-19 adding to the burden of already understaffed health system [11].

## THE FRAGILE HEALTH SYSTEM

The National Health System (NHS) of Angola covers health services comprising of both public and private sectors. NHS is supervised by the Angolan Ministry of Health. It is based on “Primary Health Care and Hospital Assistance Programme” that covers health services from primary health care to specialized care [12]. Although the public sector is free, the services available are mostly substandard with shortages of doctors, nurses, primary health care workers, medicines and digitalized health information records with undertrained doctors and nurses [13,14]. The access to health care is very limited with more than 50% of the population with no access to health care [15].

The private sector is considered to provide better health care than public sector but most private facilities are situated in the capital city making access difficult [13]. Those who can afford it use the private health facilities, while some also travel abroad to countries such as Namibia, Cuba, South Africa, Spain and Portugal for specialized private health care [13,14] but those travels have declined in the recent years due to economic crisis Angola is facing and it has become increasingly difficult due to COVID-19 travel restrictions [13].

The health infrastructures in Angola is largely underdeveloped with just one health center for 25 000 persons and one pharmacy for 22 500 persons. There is only one maternity bed for 577 births and one bed for 13 540 pediatric patients below 15 years of age. With more than 50% of its health expenditure spent on referral and central facilities, Primary Health Care (PHC) is not well developed [15]. In addition, pharmacies across Angola face shortage of drugs [14]. As of May 2020, there were only 110 Intensive Care Units (ICU) beds and 220 ventilators which is far below the required number if the COVID-19 cases peak more than 2000 [3]. In 2018, its current health expenditure was 2.55% of GDP seeing a decrease from 2.8% in the previous year with the per capita spending of US$ 87.4 in the same year (2017 = US$100.4). Sources of Health Expenditure include government transfers (41.9%), out-of-pocket-spending (36.8%), external aid (3.3%), voluntary health insurance contributions (6.7%) and others (11.2%). The government spending on health has seen a decrease in recent years with 41.9% in 2018 (2017 = 46.3%) while the out-of-pocket-spending has increased to 36.8% in the same year (2017 = 34.1%) [15].

## INFECTIOUS DISEASES

Angola, like many other developing countries with fragile health systems [16,17], has seen many infectious diseases and is vulnerable to outbreaks of malaria, yellow fever, cholera and zika and deaths from communicable diseases account for more than 50% of total death among its people [12]. Tuberculosis (TB) management in Angola has seen frequent shortages of anti TB drugs with decreased diagnosis and treatment coverage. The incidence of pulmonary TB was 182.7 per 100 000 inhabitants and prevalence (all forms of TB) was 204.1 per 100 000 inhabitants in 2017 with a record of 367 new cases of multidrug-resistant TB. Malaria remains a major public health concern accounting for approximately 35% of curable disease, 20% of hospital admissions, 40% of perinatal deaths and 25% of maternal deaths. In 2013, nearly 2.5 million cases and 6528 deaths were reported due to malaria. HIV/AIDS also remains a major public health concern in Angola with a prevalence rate of 2.1% [12]. COVID-19 that is a global public health concern [18] has added to its burden of communicable diseases and ushered in added economic burden and health challenges to its already fragile economy and health system [4].

## CONCLUSION

Benefiting from its local and global partners’ collaboration, Angola mounted an aggressive approach to contain COVID-19, helping it to keep the case load and fatality rates lower than its neighbors. However, the COVID-19 pandemic has exposed many weaknesses in health sector and the economy. Its health system is already overburdened by many infectious diseases. Thus, the government needs to reimagine its developmental policies in order to build systems resilient to health hazards and other disasters. The country must expand health infrastructures and services beyond urban reaches to rural communities where the majority of its people live. It also needs to learn from experiences of its previous infectious diseases’ outbreaks as well as the COVID-19 pandemic to build its health care capacity to provide health care for its people.
